# Network Understanding of Herb Medicine via Rapid Identification of Ingredient-Target Interactions

**DOI:** 10.1038/srep03719

**Published:** 2014-01-16

**Authors:** Hai-Ping Zhang, Jian-Bo Pan, Chi Zhang, Nan Ji, Hao Wang, Zhi-Liang Ji

**Affiliations:** 1 State Key Laboratory of Stress Cell Biology, School of Life Sciences, Xiamen University, Xiamen, Fujian, 361102, PR China; 2 Department of Chemical Biology, College of Chemistry and Chemical Engineering, The Key Laboratory for Chemical Biology of Fujian Province, Xiamen University, Xiamen, Fujian, 361005, PR China; 3 These authors contributed equally to this work.

## Abstract

Today, herb medicines have become the major source for discovery of novel agents in countermining diseases. However, many of them are largely under-explored in pharmacology due to the limitation of current experimental approaches. Therefore, we proposed a computational framework in this study for network understanding of herb pharmacology via rapid identification of putative ingredient-target interactions in human structural proteome level. A marketing anti-cancer herb medicine in China, Yadanzi (*Brucea javanica*), was chosen for mechanistic study. Total 7,119 ingredient-target interactions were identified for thirteen Yadanzi active ingredients. Among them, about 29.5% were estimated to have better binding affinity than their corresponding marketing drug-target interactions. Further Bioinformatics analyses suggest that simultaneous manipulation of multiple proteins in the MAPK signaling pathway and the phosphorylation process of anti-apoptosis may largely answer for Yadanzi against non-small cell lung cancers. In summary, our strategy provides an efficient however economic solution for systematic understanding of herbs' power.

The ancient practices of herb medicines like traditional Chinese medicine (TCM) have healed people from a variety of diseases for thousands of years. Today, herb medicines still serve as a fruitful source in treating various diseases like cancer^1^, heart failure^2^, and etc. The herb therapy in combination with conventional small molecule chemotherapy has shown promising potential in reducing drug resistance and side effects^3^. Especially, the launch of TCM modernization projects in China and abroad in recent years has made remarkable progress of herb medicine in laboratory development, ingredient profiling, and manufacture production. However, the very different theories of Western medicine versus herbal medicine have made the modernization exceedingly difficult to proceed. The limited knowledge of the TCM pharmacology has hindered its application in the mainstream medicines. Therefore, development of methodology for mechanistic study of herb medicine in molecular level that can be generally accepted by the western drug-target-disease theory is thus desired for better new drug discovery.

The non-small lung cancer (NSCLC) is the most common type of lung cancers^4^. It is only curative for a minority of patients even with incorporation of either or both radiation therapy and chemotherapy regimens. Currently, about 16 different drugs have been used in NSCLC treatment. Some of these drugs like gefitinib, erlotinib, and crizotinib were explicitly designed to inhibit primary therapeutic targets for NSCLC like the epidermal growth factor receptor (EGFR)^5^, anaplastic lymphoma receptor tyrosine kinase (ALK)^6^ and serine/threonine-protein kinase B-Raf (BRAF)^7^. Others like pemetrexed, docetaxel and gemcitabine are non-specific for NSCLC but for general cancer therapy. Unfortunately, the protein target-based chemotherapy sometimes doesn't work well due to the problem of drug resistance. For advanced NSCLC patients who can tolerate normal chemotherapy, regiments of two or more different drug types are often applied for better treatment; however, severe side effects are a non-ignorable problem. Overall, there is still in need of an efficient drug to countermine NSCLC.

In recent years, the therapeutic effects of a herb medicine Yadanzi in NSCLC treatment have been evaluated in China by many clinical cases whatever they were applied alone or in combination with other anti-cancer drugs. Yadanzi (*Fructus Bruceae*) is the dried ripe fruit of *Brucea javanica* (L.) Merr *Simaroubaceae*). As an efficient and safe traditional Chinese herb, it has saved patients from suffering of parasite infections and malarial in China for thousands of years. Recently, it also shows promising effects in cancer therapy^8^. For instance, Yadanzi recipe and its oil emulsions have been approved and commercialized in China to treat various cancer types like lung cancers, brain cancers and gastrointestinal cancers, especially middle or late stage of NSCLC^9^,^10^,^11^. Early study proposed that the oleic acids or linoleic acids, two major components of Yadanzi oil, may be responsible for the anti-cancer activities by inhibiting DNA topoisomerases I (TOP1) or II (TOP2)^12^. Some groups also proposed that Yadanzi oil could induce cancer cell death by either up-regulation of caspase-3 and caspase-9 or inhibition of NF-kappaB and Cyclooxygenase 2^13^. Moreover, some kusulactones of Yadanzi extract have been found to exhibit obvious anti-cancer activities. For example, brusatol was identified as an active compound in anti-leukaemia^14^, anti-inflammation^15^, and enhancement of neoplastic chemotherapy^16^. The anti-cancer activities were speculated to involve the nuclear factor (erythroid-derived 2)-like 2 (NFE2L2)-mediated defense mechanism^16^ and the activation and translocation of NF-kappaB into the nucleus^17^. Bruceantin is another well-studied Yadanzi ingredient that has been tested by the University of Texas system Cancer Centre in 1982 and by the Eastern Cooperative Oncology Group in 1983 for Phase II trials in treating metastatic breast carcinoma and malignant melanoma respectively^18^,^19^. It was reported that bruceantin was involved in inhibition of protein synthesis at the stage of ribosomal translation via targeting ribosome peptidyltransferase^20^. It was also found that bruceantin could induce apoptotic cell death through down-regulation of c-MYC and subsequent activation of caspase activities in mitochondria^21^. Unfortunately, the trials have been discontinued because bruceantin exhibited a relatively poor efficacy in patients and severe toxicity problems like myelosuppression and hypotension in curing bone marrow metastases. However, some researchers still believed that bruceantin should be reinvestigated as a potent agent in treatment of hematological malignancies, myeloma and other kinds of cancers^22^. Besides brusatol and bruceantin, several active ingredients like bruceine B and bruceine D were investigated for their potential activities in anti-cancers^23^,^24^ and anti-inflammation^25^. Above works, to some extent, complemented the gap of modern understanding of Yadanzi's pharmacological activities; however, the exact mechanisms in ligand-target level remain unclear. Therefore, in this study, a computational framework was introduced, which followed the empirical pharmacology theory to systematically understand herb medicine's anti-cancer activities.

## Results

### Prediction and evaluation of ingredient-target interactions

Theoretically, the common way for a drug (here, the Yadanzi ingredient) to execute its pharmacological actions is via its interaction with one or more proteins or nucleic acids (so-called therapeutic targets). In this study, total 902 distinct protein targets and 7,119 ingredient-target interactions were predicted for thirteen Yadanzi ingredients by reverse docking-based approach. Among these targets, 113 are known therapeutic targets of current marketing drugs: 54 antineoplastic/anti-cancer targets, 17 antihypertensive targets, 19 anti-inflammatory targets and etc. A complete statistics of these known therapeutic targets for Yadanzi ingredients was given in the supplementary Table S1, grouped by their potential clinical indications. Some of these clinical indications of Yadanzi ingredients have been reported, e.g. anti-inflammation activity of bruceine B, antihypertensive activity of bruceantin, and anti-cancer activities of most ingredients. Some are new roles of Yadanzi in anti-obesity, anti-rheumatism, anti-thrombus, bronchodilator, fibrinolysis, hypoglycemia, and immunomodulation.

**Figure 1:**
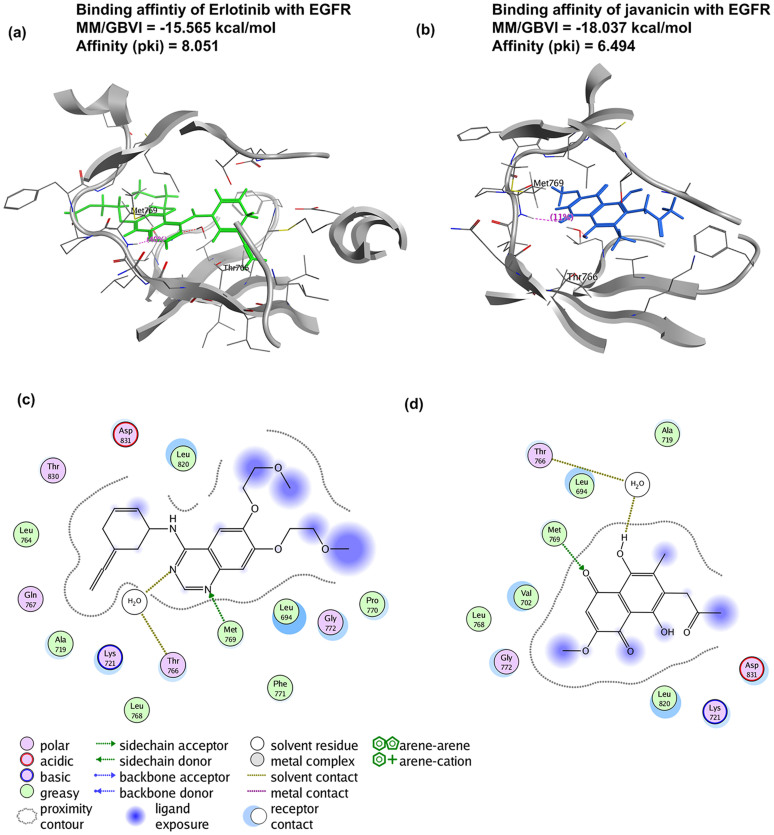
The comparative docking analysis of erlotinib-EGFR (Figure 1a) and javanicin-EGFR (Figure 1b) interactions. The key residues and forces in these two complexes are illustrated in Figure 1c and 1d respectively.

To verify these ingredient-target interactions, literature surveillance and database search were undertaken. Of 130 ingredient-disease associations derived from ingredient-target interactions (Supplementary Table S1), 10 were supported by previous experimental evidences. Unfortunately, there are few direct evidences of ingredient-target interactions. As an alternative solution, we chose 52 out of total 902 putative target proteins for comparative docking analysis, including some known therapeutic targets of current marketing drugs like the epidermal growth factor receptor (EGFR), serine/threonine-protein kinase B-Raf (BRAF), and MMPs. For instance, javanicin was predicted to interact with the human EGFR, a well-known therapeutic target of several drugs, e.g. lapatinib in curing advanced or metastatic breast cancer^26^, gefitinib and erlotinib for NSCLC therapy. As illustrated in Figure 1, both javanicin and erlotinib target EGFR at the same pocket (fragments from LEU694 to VAL702, from ALA719 to HIS781, and from HIS811 to PRO853). Previous study on erlotinib-EGFR interaction showed that the N1 of the erlotinib quinazoline accepts an hydrogen from the Met769 amide nitrogen, and the other quinazoline nitrogen atom (N3) bridges the hydrogen bond with Thr766 side chain via a water molecule (PDB ID: 1M17)^27^. Similar binding mode was also observed in the lapatinib-EGFR complex (PDB ID: 1XKK)^26^. These studies indicated that a hydrogen bond against the main chain NH of Met769 and a water-mediated hydrogen bond with Thr766 or Thr830 were critical for solid drug-EGFR binding. Comparative binding analysis showed that javanicin bound to the EGFR in the exact same mode as erlontinib, which two hydrogen bonds with the Met769 and Thr766 of EGFR were observed (Figure 1). Furthermore, the comparative binding affinities for erlotinib-EGFR and javanicin-EGFR suggested the validity of javanicin-EGFR interaction. Totally, 546 ligand-receptor interactions (including 54 drug-target interactions and 492 ingredient-target interactions) were examined, which covered 13 ingredients, 54 drugs, and 52 primary therapeutic targets (Supplementary Table S2). Of the 492 ingredient-target interactions, 145 ingredient-target interactions (covering 42 targets) show similar binding modes and comparative binding affinities (better or close MM/GBVI or pki value) to their corresponding 42 drug-target interactions (covering 27 drugs). These ingredient-target pairs were thus considered as potential strong or “true” interactions. Accordingly, 2,100 out of total 7,119 (about 29.5%) putative ingredient-target pairs were estimated to be theoretically “true” interactions. In contrast, the remaining 5,019 interactions are “weak” (they may not be false positives) compared to the known drug-target interactions which are strong in most cases. However, it is unnecessary to exclude these “weak” interactions or targets in this study, since possible add-on or synergistic effects of these interactions are often considered in conventional pharmacological studies of herbs^28^,^29^.

To further evaluate the ingredient-target interactions, additional pharmacophore analysis was undertaken as well upon the above 52 selective target proteins. For each selective target, the drugs and the predicted docking ingredients (Supplementary Table S3) were structurally aligned to seek whether common features existed leading to similar target-binding. It was found that all 52 drug-ingredient pairs (corresponding to 52 target proteins) contain at least one common pharmacophore feature, and 37 out of 52 proteins share at least three common pharmacophore features (varied by drug-ingredient pairs) (Supplementary Table S4). For instance, four common pharmacophore features were identified between Yadanzi ingredients and their corresponding marketing drugs when binding to EGFR (Supplementary Figure S1), among which the pseudo aromatic rings acceptors (Feature 2) were found to be crucial in drug-protein interaction according to former research^26^,^27^. These consensus pharmacophore features further validated the predicted ingredient-target interactions in a way that is completely different from the docking-based approaches.

It is worthy of mention that some ingredients show much better binding affinities than marketing drugs against their therapeutic targets. For example, bruceine C's binding affinity against the nitric oxide synthase 2 (NOS2) was estimated to about seven times of its corresponding drug dexamethasone, an anti-inflammatory and immunosuppressant. Bruceoside E's binding affinity was estimated to about seventeen times of marimastat against its primary target MMP1. A list of such ingredient-target interactions were given in Supplementary Table S5. These findings hint some novel clinical indications of Yadanzi ingredients. Further experimental validations are desired.

### The predicted targets of Yadanzi active ingredients

According to their similarities in QSAR properties, the thirteen ingredients of Yadanzi were categorized into 5 groups: Group I (bruceine_b, bruceine_d, bruceine_g, bruceine_i, brusatol), Group II (bruceantin, bruceine_c, bruceine_e, bruceoside_e), Group III (bruceantinol, bruceoside_a), Group IV (bruceoside_b) and Group V (javanicin) (Supplementary Figure S2). It seems that ingredients in the same group intend to have similar pharmacological activities (Supplementary Table S1).

Brusatol was predicted to target 464 proteins, among which 46 are known targets of marketed drugs in cancer therapy (Supplementary Table S1), including the matrix metalloproteinase-1 (MMP1) and MMP8 for lung cancer^30^,^31^,^32^, MMP7 and chromodomain-helicase-DNA-binding protein 1 (CHD1) for primary breast cancer and prostate cancer^33^, TOP1 for metastatic colorectal cancer and extensive small cell lung cancer^34^,^35^. According to our comparative docking analysis, brusatol shows comparative or better affinity with these five proteins than their corresponding marketed drugs (Supplementary Table S5&S2). Of the 477 putative protein targets identified for bruceantin in this study, 36 are known therapeutic targets of current marketing anti-cancer drugs (Supplementary Table S1). Among these anti-cancer targets, 3-phosphoinositide-dependent protein kinase-1(PDPK1), MMP1, MMP2, MMP3, nitric oxide synthase (NOS2), and inosine-5′-monophosphate dehydrogenase 2 (IMPDH2) showed similar binding affinity with bruceantin as that of some marketing drugs (Supplementary Table S2). Literature surveillance indicated that MMP1, MMP2 and MMP3 were responsible for various cancers including lung cancer^30^,^31^,^32^; NOS2 was involved in wound healing, angiogenesis, and carcinogenesis; IMPDH2 was a therapeutic target of marketing drug mycophenolic acid for immunosuppressance and anti-proliferation. It is also impressing that 11 out of 477 proteins were recognized primary targets for improving hypertensive situations. Many of these 11 proteins are either involved in the vascular smooth muscle contraction pathway or in the renin-angiotensin system pathway. These findings, to some extent, explain the dose-dependent side effect of hypotension induced by bruceantin. A complete therapeutic target list of Yadanzi ingredients was given in the Supplementary Table S1.

**Figure 2:**
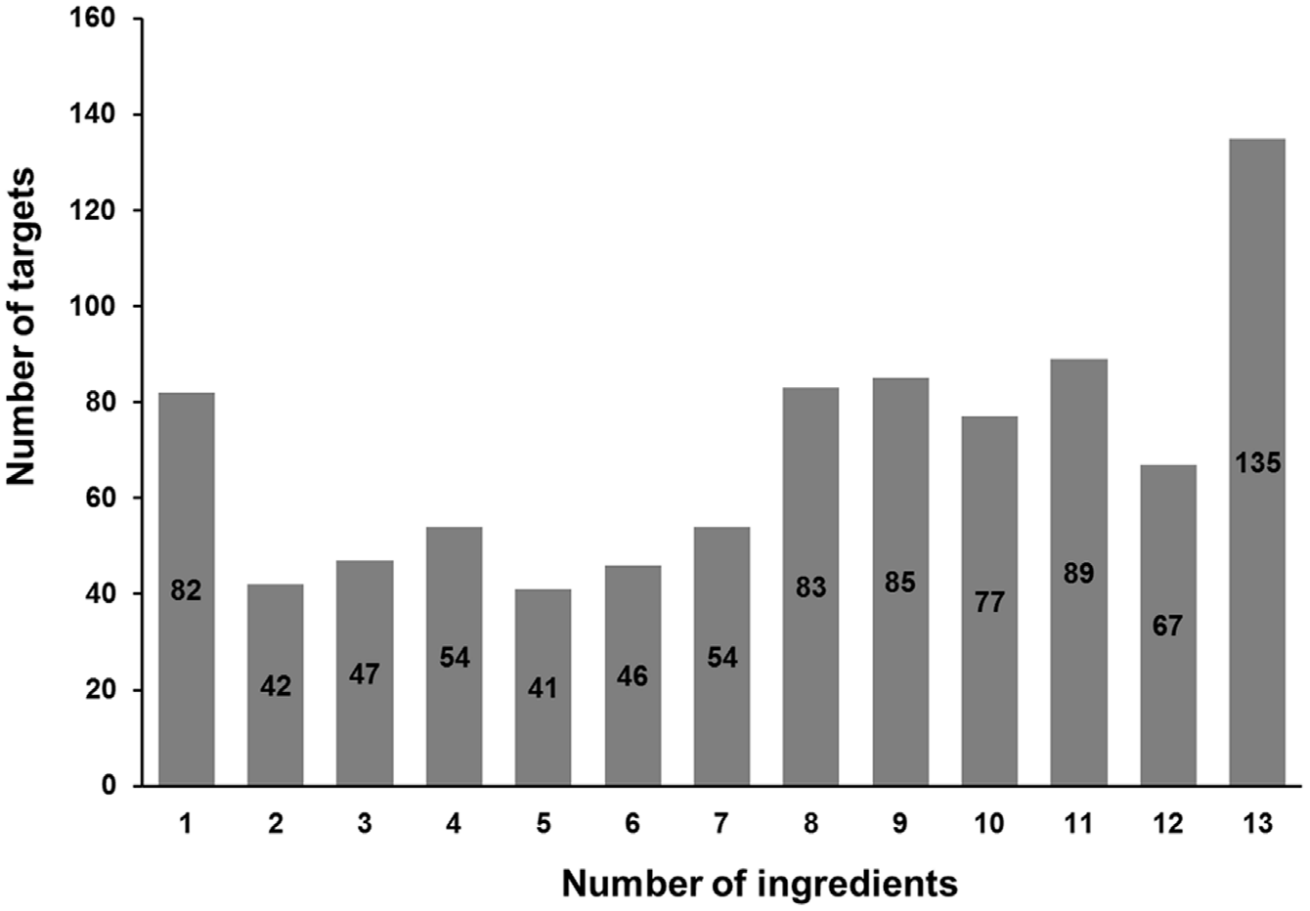
The functional promiscuities of Yadanzi ingredients and their putative targets.

It is interesting but not surprising that functional promiscuity was commonly observed for both Yadanzi ingredients and their putative targets. Of 902 total distinct protein targets predicted in this study, 778 were shared by at least three ingredients, and 135 were common in all thirteen ingredients (Figure 2). The functional promiscuity of Yadanzi's ingredients and their corresponding targets was also reflected by their associations with various diseases, not only conventional parasite infections and malarial but also various types of cancers. Furthermore, an additional pathway enrichment analysis was performed, using the NIH DAVID Tools^36^, against a background population of all KEGG pathway-annotated proteins. The result showed that 603 predicted targets were annotated in KEGG pathways, and enriched in 14 cancer pathways (Supplementary Table S6). For instance, 17 predicted targets were identified in the pathway of non-small cell lung cancer (hsa05223) with a fold enrichment factor of 2.65 (p value: 3.39 × 10^−4^), which indicated the very association of Yadanzi with NSCLC comparing to the background pathways. Besides NSCLC, some cancers like melanoma were also found highly associated with Yadanzi. Actually, some of them have been clinically treated or tested by Yadanzi or its ingredients^18^.

### The possible mechanisms of Yadanzi's anti-NSCLC activity

**Figure 3:**
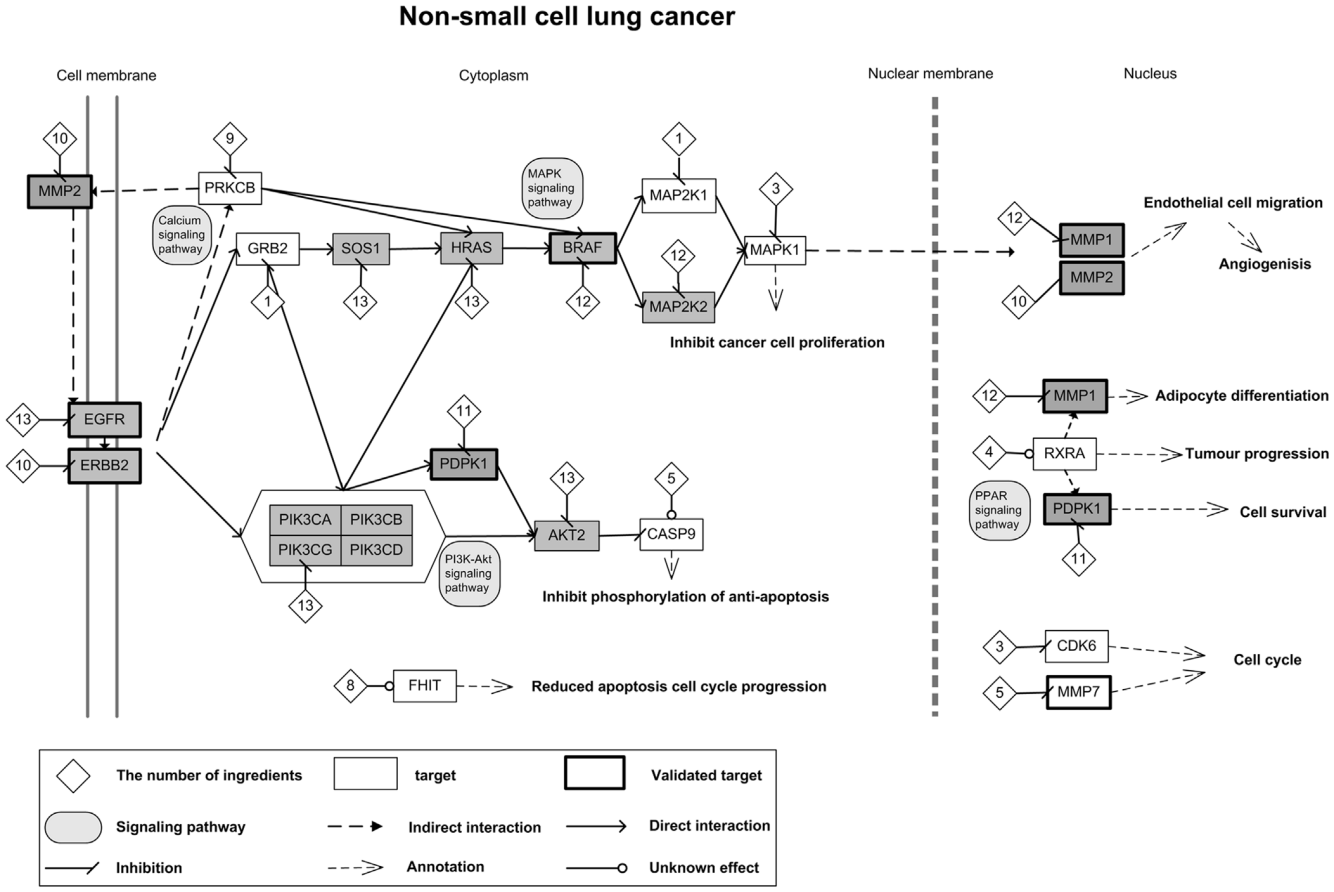
The diagram of possible molecular mechanism underlying Yadanzi's anti-NSCLC activity. The square stands for the putative target protein of Yadanzi ingredients predicted in this study (the proteins which targeted by equal to or more than 10 ingredients are marked gray). The thicker protein block indicates at least one ingredient-target interaction has been validated by previous experimental evidences or computational simulation in this study. The diamond stands for ingredient, and the number in the diamond is the number of ingredients binding to the corresponding protein. The actions (inhibition of activation) of ingredient-protein interactions were determined by comparative docking analysis.

Yadanzi ingredients were predicted to interact with 902 distinct targets using the reverse docking approach. By mapping these predicted targets into the KEGG pathway of NSCLC (hsa05223), 17 NSCLC-associated proteins were identified. In addition, three putative targets (MMP1, MMP2, and MMP7) of marketing anti-cancer drugs were reported to be associated with NSCLC. These 20 NSCLC-associated proteins included some known therapeutic targets like EGFR and BRAF as well as some putative targets like HRAS and AKT2 that haven't been used in NSCLC treatment. Their connections and impacts on NSCLC were illustrated in Figure 3.

As shown in the network (Figure 3), the Yadanzi may realize its anti-NSCLC activity mainly via two routes: One was through the disturbance of MAPK signaling pathway, which leaded to blockage of the NSCLC cell proliferation. The other was via inhibition of the phosphorylation processes of anti-apoptosis effects. Both these two routes were regulated by the upstream ErbB signaling pathway, via either of both of EGFR and the receptor tyrosine-protein kinase erbB-2 (ERBB2, also named HER2). Of the ten putative targets in the first route, EGFR^9^,^37^, ERBB2^38^,^39^, BRAF^40^ and mitogen-activated protein kinase 1 (MAPK1, also known as ERK2)^41^,^42^ were known primary anti-tumor targets of several marketing drugs; growth factor receptor-bound protein 2 (GRB2), Harvey rat sarcoma viral oncogene homolog (HRAS), protein kinase C, beta (PRKCB) and mitogen-activated protein kinase kinase 1 (MAP2K1) were research targets for countermining cancers; SOS1 and MAPK2K2 were putative targets that haven't been reported for cancer therapy yet. It was noticed that three proteins (EGFR, HRAS, and SOS1) in this route were predicted to interact with all thirteen ingredients and two proteins (BRAF and MAPK1) were targeted by twelve ingredients.

Other than inhibition of cancer cell proliferation, the Yadanzi may stop tumor progress by intervening phosphorylation process of anti-apoptosis effects. Of the six proteins/complexes in this route, EGFR, ERBB2, and the 3-phosphoinositide-dependent protein kinase 1 (PDPK1)^43^,^44^ were known primary target of marketing anti-cancer drugs. The other three proteins, phosphatidylinositol-4,5-bisphosphate 3-kinase, catalytic subunit gamma (PIK3CG), v-akt murine thymoma viral oncogene homolog 2 (AKT2) and caspase 9, apoptosis-related cysteine peptidase (CASP9) were all involved in tumorigenesis or cancer progress^45^,^46^,^47^. Among these six proteins, EGFR, PIK3CG and AKT2 were targeted by all 13 Yadanzi ingredients; ERBB2, PDPK1 and CASP9 were targeted by 10, 11 and 5 ingredients, respectively. Interestingly, these two routes were not isolated in anti-NSCLC that they have cross-talks bridged by EGFR, ERBB2, GRB2, HRAS and PIK3. Besides, Yadanzi may intercept NSCLC tumorgenesis by disturbing a number of pathways like cell cycle via CDK6, MMP7 and FHIT, PPAR signaling pathway via RXRA, PDPK1 and MMP1, calcium signaling pathway via PRKCB, and etc. These proteins or pathways were peripheral of the above two routes. However, interference with these targets/pathways would more or less contribute to Yadanzi's anti-NSCLC activities.

## Discussion

Thousands of years' clinical practice has proved the power of herbs in protecting people from various diseases. Nowadays, active compounds were extracted and purified from herbs or natural products for further new drug discovery. Unfortunately, many drug candidates developed in this way often fail due to inadequate efficacy or severe toxicity in clinical trials. These failures are mainly caused by lacking a panorama view of how herb ingredients together intervene in the pathogenetic processes and realize their therapeutic effects. This may include a number of ligand (ingredient)-receptor (protein target) interactions, determination of which would be a rational shortcut to understand herb and properly use it. On the other side, most diseases are the consequence of multiple pathogenetic processes. Each of these processes involves a bundle of molecules, which communicate with each other and extend to form an incredible complex molecular network. During the past several decades, the thought of designing exquisitely selective ligands against individual drug target to rectify the pathogenetic condition back to the normal state has been predominant in drug discovery^48^. In most cases, drugs designed in this theory exhibit expected therapeutic effects. However, current advances in systems biology suggest that such strategy is sometimes no longer applicable in countermining complex diseases like cancers^49^, since a blocked pathogenetic process can be complemented by the others. Many effective drugs for cancers, psychiatry and infectious diseases actually act on multiple targets rather than single one^50^. Alternatively, advanced chemotherapies like multi-target drug, combinational drug formula and cocktail therapy were thus proposed in recent years to interfere multiple pathogenetic processes simultaneously for better treatment. To develop a robust advanced chemotherapy formula, a relatively complete profile of drug-target interactions is expected, which is hard to achieve by traditional molecular technologies.

Here, we proposed a computational strategy, which is able to screen the available human protein structures for putative ligand-receptor interactions and connect them into network for better investigation of molecular mechanisms underlying herbs' pharmacological activities (Supplementary Table S6). As shown in the network (Figure 3), many NSCLC-associated proteins were targeted by multiple Yadanzi ingredients; some are even targeted by all thirteen ingredients. The promiscuity of putative targets matches current pharmacological theory of herb medicine that “multi-ingredient against multi-target with comparably low-affinity binders”^28^,^51^. Acting on the same targets greatly enhances the binding opportunity of Yadanzi ingredients and thus makes the herb treatment more effective, likely via mechanisms of add-on effects or even synergistic effects. On the other side, the versatility of ingredient by targeting on multiple proteins in the disease-related pathways, to some extent, guarantees the effective and stable herb therapy regardless of genetic variations on single target. It may explain why Yadanzi, as an herb itself, has much better efficacy than its single ingredient like bruceantin in treating various cancers. In the case of NSCLC (Figure 3), 13 out of total 20 NSCLC-associated proteins were targeted by bruceantin. These 13 proteins don't include some crucial proteins in NSCLC development like CASP9, CDK6, GRB2, FHIT, MAPK1, MAP2K1 and MMP7. Interception of several protein nodes may not efficiently block NSCLC development. Previous network biology analysis proposed that deleting single node has little effect on disease network as existence of alternative compensatory signaling routes. Hence, modulating multiple key node proteins may be required to efficiently break robustness of disease network^28^,^51^. In the paths associated with NSCLC, some proteins, e.g., AKT2, EGFR, ERBB2, HRAS, and MAPK1, are likely key node proteins, deletion of which may lead to shutdown of the sub-routes. It was observed that many of these key node proteins were heavily regulated by Yadanzi ingredients, for instance, EGFR and AKT2 were targeted by all 13 ingredients. These may partially explain the efficacy of Yadanzi in countermining cancers.

Worthy of mention, Yadanzi ingredients were predicted to target additional 882 proteins (many of the ingredient-target interactions may be relatively “weak”) besides the 20 NSCLC-associated proteins. These “weak” ingredient-target interactions are involved in different cellular processes like metabolisms (nitrogen metabolism, retinol metabolism, drug metabolism, and etc) and signal transductions (Wnt signaling pathway, insulin signaling pathway, MAPK signaling pathway, and etc), forming an incredibly complex molecular network that more or less connects with NSCLC development and spread as well as other pathogenesis.

The computational approach introduced in this study provides a much broader vision of pharmacology than traditional molecular approaches; however, at the same time, the large numbers of ligand-receptor interactions sometimes make investigation defocused. The docking-based approach adopted in this study itself is still far from perfectness. Its robustness may be affected by aspects of: (1) limitation of protein structures available in PDB database, (2) difficulty in setting a threshold free binding energy for proper assignment of “real” ligand-receptor interaction, and (3) short of a reliable method for determining physiological actions of ligand-receptor binding (inhibition or activation). These shortcomings are hard to be completely solved so far; but can be improved, to some extent, by incorporating additional algorithms like comparative docking analysis and pharmacophore analysis in this study. Besides, many aspects of herb pharmacology haven't been discussed, including the pharmacokinetics, exposure-response relation, immunological response, genetic variations and so on. These aspects somehow determine the successfulness of small molecule drug discovery, which should be considered in modernization of herb medicine as well.

Nevertheless, the strategy of ligand-target interaction network analysis suggests a systematic way in probing molecular mechanisms underlying complex pharmacological or toxicological events which are difficult to be revealed by current experimental approaches. It is an audacious trial in system pharmacology/toxicology, which opens a window to spy upon the molecular secret of herbs' power in a way that recognized by nowadays medicine theory. In the meanwhile, this strategy proposes a feasible and economic method in early stage of novel drug discovery from herbs or natural products. It is no longer constrained to the conventional theory of “one drug, one target, and one disease”, but reviewing the impact of the candidate compound on all possible pathogenetic processes via interaction with multiple proteins, so-called “system pharmacology or network pharmacology”. Novel findings of ingredient-protein target interactions also inspire us to mine the herb nature for better therapy.

## Methods

### Information & QSAR classification of Yadanzi active ingredients

The information of Yadanzi ingredients was derived from the TCM database (http://bidd.nus.edu.sg/group/TCMsite/query.aspx)^52^. Besides the oleic acids and linoleic acids, there are thirteen major active ingredients of Yadanzi. The structures of these ingredients were downloaded from the PUBCHEM database (http://pubchem.ncbi.nlm.nih.gov/)^53^ and further refined by energy minimization without changing their chirality (Supplementary Figure S2). The quantitative structure-activity relationship (QSAR) properties of these thirteen ingredients were computed using the local version of software MODEL (Molecular Descriptor Lab)^54^, by which 1,559 distinct descriptors, including number of atoms, total absolute atomic charge, maximum positive charges, topological radius, and average molecular weight were selected. According to their similarity in QSAR properties, the thirteen ingredients were further clustered by Hierarchical Clustering module of the MATLAB into groups.

### Identification of putative ingredient-target interactions

The putative protein targets of Yadanzi's ingredients were identified by simulation of ligand-receptor binding using the reverse docking software INVDOCK^55^. INVDOCK is a ligand-protein reverse docking algorithm, which conducts computer-automated search of potential protein targets of a small molecule by attempting to dock it into the cavities of proteins. The target prediction was demonstrated in a high-throughput manner that each Yadanzi ingredient was traversed all human protein cavities in PDB structure database (http://www.rcsb.org/)^56^ (by September 2012) for possible binding. The algorithm of INVDOCK has been well described in previous study^55^. Putative protein targets were selected based on an implemented scoring scheme that performs binding competitive analysis in addition to the evaluation of molecular mechanics ligand-protein interaction energy^57^. An empirical threshold score of smaller than −20 was adopted for positive assignment of drug-target interactions. The docked protein list for each ingredient was further refined by removing redundant targets with multiple PDB IDs so that only one representative was retained.

### Comparative docking analysis

Selective ingredient-target interactions were verified by comparing their binding affinities to corresponding marketing drug-target interactions against the same therapeutic protein targets. The information of marketing drugs and their therapeutic targets were acquired from the DRUGBANK database (http://www.drugbank.ca/)^58^. The 3D structures of the drugs were downloaded from DRUGBANK, PUBCHEM or PDB databases, and further refined by energy minimization without changing their chirality. For each ingredient-drug pair, same active pocket site on the protein target was chosen for comparative binding affinity analysis. The active pocket sites were either extracted from available drug-target complexes in PDB or derived from previous literatures. The flexible docking was undertaken using the Simulations/Dock module of the commercial Molecular Operating Environment (MOE) software. Solvation effects were calculated using the reaction field functional form for the electrostatic energy term. A theoretical “strong” ingredient-target interaction was declared by satisfying following two conditions: similar binding pattern (same active site, similar key residues and interacting forces) and comparative binding affinity. The Molecular Mechanics Generalized Born Interaction energy (MM/GBVI) and affinity (pki) of the refined ligand-receptor complexes were further estimated by the Ligand Explorer model (LigX) of MOE software. The physiological action of ingredient-target interaction, activation or inhibition, was referred to its corresponding drug-target interaction.

### Pharmacophore analysis

The pharmacophore analysis were undertaken to seek the common physiochemical and structural features between marketing drugs and Yadanzi ingredients against their corresponding common protein target (the active binding site). It was assumed that if the ingredients shared more pharmacophore features with the marketing drugs, they are more likely to have similar protein binding ability. Therefore, for a given protein target (Supplementary Table S3), the ingredients and the drugs were first structurally aligned using the Flexible Alignment module of MOE. This followed by the pharmacophore consensus calculation to identify putative pharmacophore feature groups using the Pharmacophore module of MOE software. For each pharmacophore feature group against a respective target, its consensus was measured by equation (1). 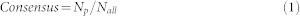
N_p_ is the number of ingredients that contain this pharmacophore group, and N_all_ is the number of total ingredients that bind to the respective target protein.

### Construction of ingredient-target interaction network

For better illustration of the molecular mechanisms underlying Yadanzi's pharmacological activities, the ingredient-target interaction networks were constructed as following: First, for each Yadanzi's pharmacological activity, e.g. anti-NSCLC, the activity-associated targets were determined by superimposing the predicted non-redundant putative targets of Yadanzi ingredients against the activity-associated pathways recorded in KEGG database (http://www.genome.jp/kegg/)^59^. In addition, the activity-associated targets were also collected from literature. Then, the connections between the activity-associated targets were mainly derived from the KEGG database as well as some protein-protein interaction databases like STRING (http://string-db.org/). Upon these protein connection data, a network of ingredient-target interaction was then constructed manually by referring to the KEGG pathway.

## Author Contributions

Z.L.J. formulated the idea of the paper and supervised the research. H.P.Z., J.B.P., C.Z., N.J. and H.W. performed the research. H.P.Z., J.B.P. and Z.L.J. wrote the paper.

## Supplementary Material

Supplementary Information supplementary material
