# Supplementary material for: Network Understanding of Herb Medicine via Rapid Identification of Ingredient-Target Interactions
Source: Sci Rep. 2014 Jan 16;4:3719. doi: 10.1038/srep03719 (PMC3893644; doi:10.1038/srep03719)
Supplement: Supplementary Information — supplementary material [file srep03719-s1.pdf]

Network Understanding of Herb Medicine via Rapid Identification of Ingredient-Target Interactions

Hai-Ping Zhang [1,#], Jian-Bo Pan [2,#], Chi Zhang [1], Nan Ji [1], Hao Wang [2], Zhi-Liang Ji [1, 2, *]

[1] State Key Laboratory of Stress Cell Biology, School of Life Sciences, Xiamen University, Xiamen, Fujian, 361102, PR China

[2] Department of Chemical Biology, College of Chemistry and Chemical Engineering, The Key Laboratory for Chemical Biology of Fujian Province, Xiamen University, Xiamen, Fujian, 361005, PR China

*Corresponding author: Tel.: 86-0592-2182897; Fax: 86-0592-2182897; E-mail: appo@xmu.edu.cn

# These two authors contributed equally to this work.

Figure S1. The pharmacophore analysis of drugs and Yadanzi ingredients against the common protein target EGFR. The pharmacophore groups were determined using the Pharmacophore module of the Molecular Operating Environment (MOE) software.

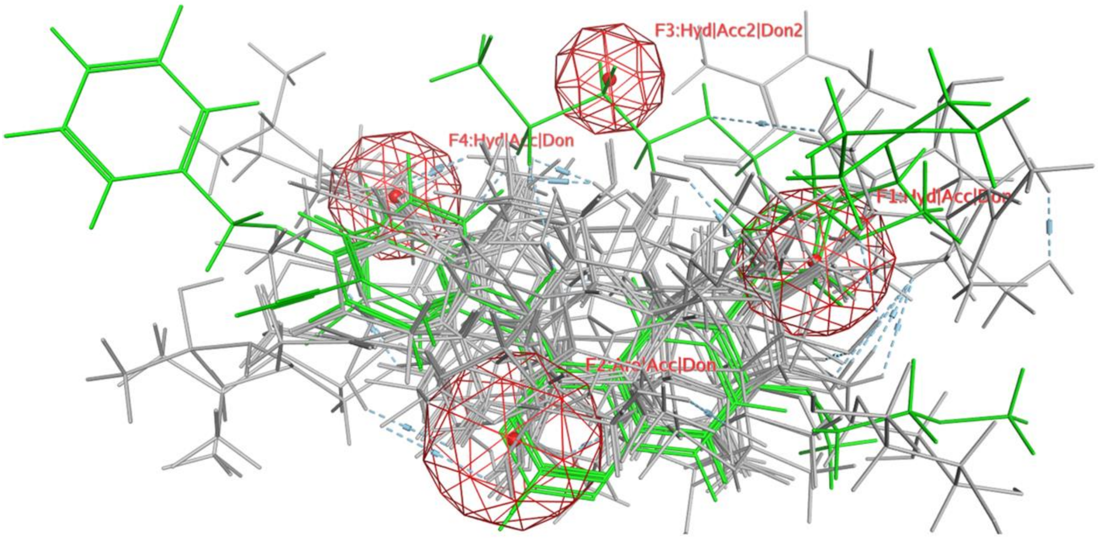

Hyd|ACC|Don	H-bond Acceptor | Donor
F	Pharmacophore Feature
Aro|ACC|Don	Aromatic and pseudo aromatic rings Acceptor | Donor
Hyd|ACC|Don	H-bond Acceptor Projection | Donor projection

Figure S2. The structure of the thirteen ingredients of Yadanzi, grouped by their similarities in QSAR properties.

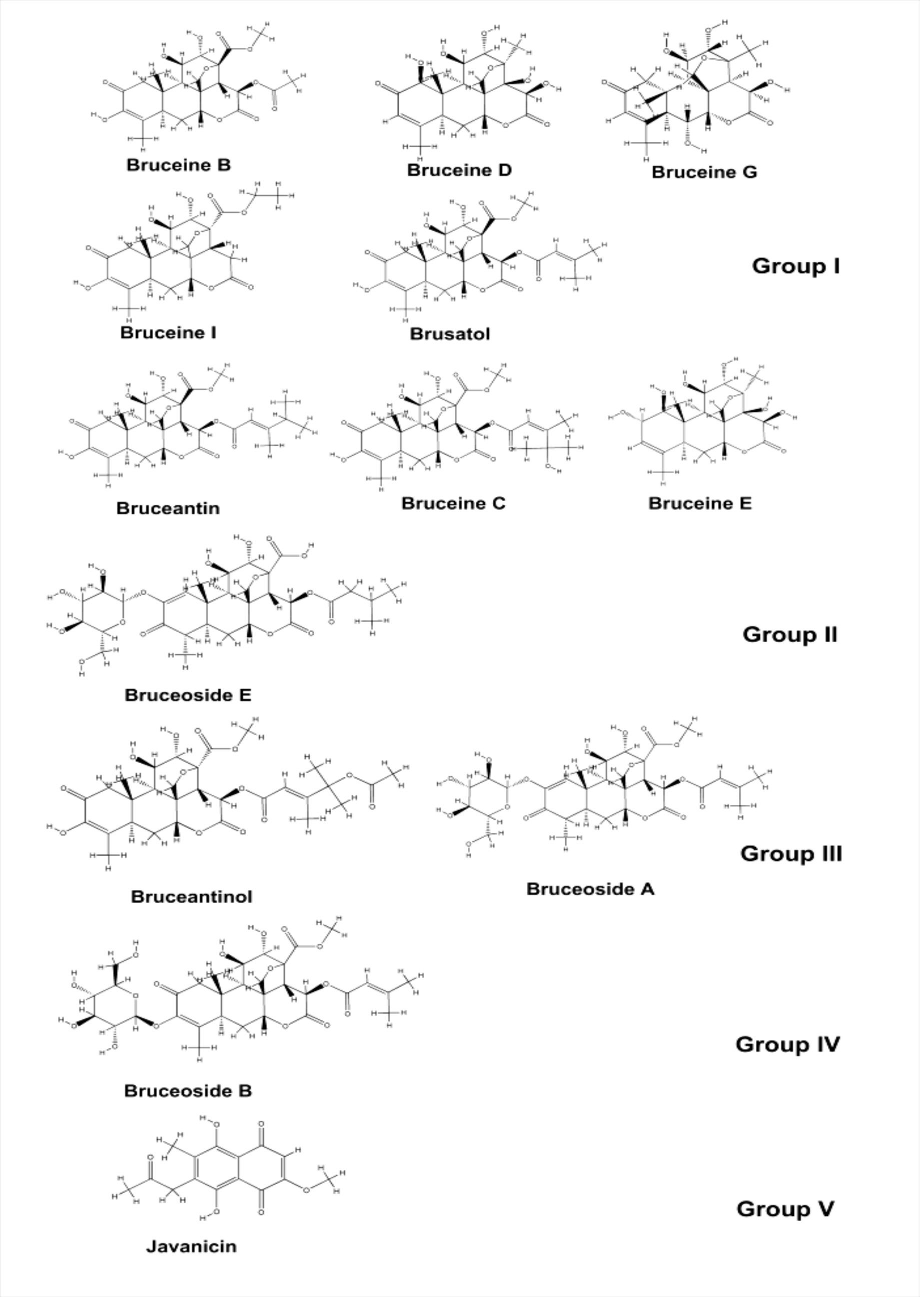

Table S1. Putative targets of Yadanzi thirteen ingredients and their potential clinical indications.

Therapeutic Activities [a]	Group I					Group II				Group III		Group IV	Group V	Sum
	Bruceine B	Bruceine D	Bruceine G	Bruceine I	Brusatol	Bruceantin	Bruceine C	Bruceine E	Bruceoside E	Bruceantinol	Bruceoside A	Bruceoside B	Javanicin	
Anti-hypertension	15 CA1, MMP2, PPARD, CA2, MME, ACE, REN, CA12, PDE4B, NR3C2, TNNC1, AKR1C3, PDE4D, CALM1, PLAT	16 CA1, MMP2, PPARD, CA2, MME, ACE, REN, AOC3, AVPR1A, PDE4B, NR3C2, TNNC1, AKR1C3, PDE4D, CALM1, PLAT	15 CA1, MMP2, PPARD, CA2, MME, ACE, REN, NR3C2, CA12, AVPR1A, PDE4B, TNNC1, PDE4D, CALM1, PLAT	15 CA1, MMP2, PPARD, CA2, MME, ACE, REN, NR3C2, CA12, AVPR1A, PDE4B, TNNC1, PDE4D, CALM1, PLAT	13 CA1, MMP2, PPARD, CA2, MME, ACE, REN, PDE4B, NR3C2, TNNC1, PDE4D, CALM1, PLAT	11 CA1, MMP2, PPARD, CA2, MME, ACE, REN, PDE4B, PDE4D, CALM1, PLAT	13 CA1, PPARD, CA2, MME, ACE, REN, AVPR1A, PDE4B, NR3C2, TNNC1, PDE4D, CALM1, PLAT	16 CA1, MMP2, PPARD, CA2, MME, ACE, REN, AOC3, NR3C2, CA12, AVPR1A, TNNC1, AKR1C3, PDE4D, CALM1, PLAT	7 MMP2, PPARD, MME, ACE, REN, PDE4D, CALM1	10 CA1, MMP2, CA2, MME, ACE, REN, PDE4B, PDE4D, CALM1, PLAT	4 ACE, REN, PDE4D, CALM1	6 CA2, ACE, REN, PDE4D, CALM1, PLAT	15 CA1, MMP2, PPARD, CA2, MME, ACE, REN, NR3C2, CA12, AVPR1A, TNNC1, AKR1C3, PDE4D, CALM1, PLAT	17
Anti-inflammation	17 NR3C1, RXRA, PPARD, PDXK, FDPS, PLA2G2A, PDXP, AKR1B1, ALDH7A1, HSP90AA1, CBS, GLO1, NR3C2, PGR, FGF1, NOS2, PPARG	18 HSP90AA1, NR3C1, RXRA, PPARD, PDXK, FDPS, PLA2G2A, PDXP, AKR1B1, ALDH7A1, CBS, GLO1, FGF1, AR, NR3C2, PGR, NOS2, PPARG	18 NR3C1, RXRA, PPARD, PDXK, FDPS, PLA2G2A, PDXP, AKR1B1, ALDH7A1, HSP90AA1, CBS, GLO1, FGF1, NR3C2, AR, PGR, NOS2, PPARG	18 NR3C1, RXRA, PPARD, PDXK, FDPS, PLA2G2A, PDXP, AKR1B1, ALDH7A1, HSP90AA1, CBS, GLO1, DHODH, NR3C2, AR, PGR, FGF1, NOS2	16 NR3C1, RXRA, PPARD, PDXK, FDPS, PLA2G2A, PDXP, AKR1B1, ALDH7A1, HSP90AA1, CBS, GLO1, NR3C2, PGR, NOS2, PPARG	14 NR3C1, RXRA, PPARD, PDXK, FDPS, PLA2G2A, PDXP, AKR1B1, ALDH7A1, HSP90AA1, CBS, GLO1, PGR, NOS2	14 NR3C1, RXRA, PPARD, PDXK, FDPS, PLA2G2A, AKR1B1, HSP90AA1, CBS, GLO1, FGF1, NR3C2, NOS2	18 NR3C1, RXRA, PPARD, PDXK, FDPS, PLA2G2A, PDXP, AKR1B1, ALDH7A1, HSP90AA1, CBS, GLO1, FGF1, NR3C2, AR, PGR, NOS2, PPARG	12 PPARD, PDXK, PLA2G2A, PDXP, AKR1B1, ALDH7A1, HSP90AA1, CBS, GLO1, RXRA, NOS2, PPARG	13 NR3C1, RXRA, PDXK, FDPS, PLA2G2A, PDXP, AKR1B1, ALDH7A1, HSP90AA1, CBS, GLO1, PGR, NOS2	11 PDXK, PDXP, AKR1B1, ALDH7A1, HSP90AA1, CBS, GLO1, RXRA, NOS2, PPARG	5 FDPS, CBS, GLO1, NOS2, PPARG	18 NR3C1, RXRA, PPARD, FDPS, PLA2G2A, PDXP, AKR1B1, ALDH7A1, HSP90AA1, CBS, GLO1, DHODH, NR3C2, AR, PGR, FGF1, NOS2, PPARG	19
Antineoplastion or Anticancer	44 ABL1, AKR1B1, ATIC, BRAF, C1R, CHD1, CSF1R, DCK, DHFR, EGFR, ERBB2, ESR1, ESRRG, F2, FDPS, GART, GSR, HDAC8, HMGCR, HPRT1, IMPDH2, ITGAL, KDR, KIT, LDLR, MAPK1, MMP1, MMP10, MMP12, MMP2, MMP3, MMP7, MMP8, NOS2, PGR, PLA2G2A, PNP, POLB, PPARD, RARA,	47 ABL1, AKR1B1, AR, ATIC, BRAF, C1R, CHD1, CSF1R, DCK, DHFR, EGFR, ERBB2, ESR1, ESRRG, F2, FDPS, FSHR, GART, GSR, HDAC8, HMGCR, HPRT1, IMPDH2, ITGAL, KDR, KIT, LDLR, MMP1, MMP10, MMP12, MMP2, MMP3, MMP7, MMP8, NOS2, NR3C1, PGR, PLA2G2A,	44 ABL1, AKR1B1, AR, ATIC, BRAF, C1R, CHD1, CSF1R, DCK, DHFR, EGFR, ERBB2, ESRRG, F2, FDPS, FSHR, GART, GSR, HDAC8, HMGCR, HPRT1, IMPDH2, ITGAL, LDLR, MMP1, MMP10, MMP12, MMP2, MMP3, MMP8, MMP9, NOS2, NR3C1, PGR, PLA2G2A, PNP, POLB, PPARD, RXRA, SHMT1, SIRT5, TOP1, TYMS, VDR	48 ABL1, AKR1B1, AR, ATIC, BRAF, C1R, CHD1, CSF1R, DCK, DHFR, DHODH, EGFR, ERBB2, ESR1, ESRRG, F2, FDPS, FSHR, GART, GSR, HMGCR, HPRT1, IMPDH2, ITGAL, KDR, KIT, LDLR, MMP1, MMP10, MMP12, MMP2, MMP3, MMP7, MMP8, NOS2, NR3C1, PGR, PLA2G2A,	46 ABL1, AKR1B1, ATIC, BRAF, C1R, CHD1, CSF1R, DCK, DHFR, EGFR, ERBB2, ESR1, ESRRG, F2, FDPS, FSHR, GART, GSR, HMGCR, HPRT1, IMPDH2, ITGAL, KIT, MAPK1, MMP1, MMP10, MMP12, MMP13, MMP2, MMP3, MMP7, MMP8, NOS2, NR3C1, PGR, PLA2G2A, PNP, POLB, PPARD, RARA, RXRA, SHMT1, SIRT5, TOP1, TUBG1, TYMS	36 ABL1, AKR1B1, ATIC, BRAF, CHD1, CSF1R, DCK, DHFR, EGFR, ERBB2, ESR1, ESRRG, F2, FDPS, GART, GSR, HMGCR, IMPDH2, ITGAL, KDR, MMP1, MMP10, MMP12, MMP2, MMP3, MMP8, NOS2, PGR, PLA2G2A, PNP, POLB, PPARD, RXRA, SIRT5, TOP1, TYMS	37 ABL1, AKR1B1, ATIC, BRAF, CHD1, CSF1R, DHFR, EGFR, ERBB2, ESR1, ESRRG, F2, FDPS, FSHR, GART, GSR, HMGCR, HPRT1, IMPDH2, KDR, LDLR, MAPK1, MMP1, MMP10, MMP12, MMP13, MMP3, NOS2, PLA2G2A, PNP, POLB, PPARD, RARA, RXRA, SHMT1, SIRT5, TOP1,	44 ABL1, AKR1B1, AR, ATIC, C1R, CHD1, CSF1R, DCK, DHFR, EGFR, ERBB2, ESR1, ESRRG, F2, FDPS, FSHR, GART, GSR, HDAC8, HMGCR, HPRT1, IMPDH2, ITGAL, KDR, KIT, LDLR, MMP1, MMP10, MMP12, MMP2, MMP3, MMP8, NOS2, NR3C1, PGR, PLA2G2A, PNP, POLB, PPARD,	26 ABL1, AKR1B1, BRAF, CHD1, DHFR, EGFR, ESR1, ESRRG, F2, FSHR, GART, HMGCR, IMPDH2, KDR, KIT, MMP1, MMP10, MMP12, MMP2, NOS2, PLA2G2A, PNP, POLB, PPARD, RXRA, TOP1	29 ABL1, AKR1B1, ATIC, BRAF, CHD1, DHFR, EGFR, ESR1, F2, FDPS, GART, HMGCR, IMPDH1, IMPDH2, KDR, LDLR, MMP1, MMP12, MMP2, MMP3, NOS2, PGR, PLA2G2A, PNP, POLB, RARA, RXRA, TOP1, TYMS	16 ABL1, AKR1B1, ATIC, BRAF, CHD1, EGFR, F2, FSHR, GART, HMGCR, IMPDH2, MMP12, NOS2, PNP, POLB, RXRA, TOP1	18 ABL1, ATIC, BRAF, CHD1, EGFR, ERBB2, ESR1, F2, FDPS, GART, HMGCR, KDR, KIT, MMP1, MMP12, NOS2, POLB, RARA, TOP1, TUBG1	47 ABL1, AKR1B1, AR, ATIC, BRAF, C1R, CHD1, CSF1R, DCK, DHFR, DHODH, EGFR, ERBB2, ESR1, ESRRG, F2, FDPS, FSHR, GART, GSR, HDAC8, HMGCR, HPRT1, IMPDH2, ITGAL, KIT, LDLR, MMP1, MMP10, MMP12, MMP13, MMP2, MMP3, MMP7, MMP8, MMP9, NOS2, NR3C1, PGR, PLA2G2A, PNP, POLB, PPARD, RXRA, SHMT1, SIRT5, TOP1, TYMS, VDR	54

	RXRA, SHMT1, SIRT5, TOP1, TYMS	PNP, POLB, PPARD, RARA, RXRA, SHMT1, SIRT5, TOP1, TYMS		PNP, POLB, PPARD, RARA, RXRA, SHMT1, SIRT5, TOP1, TYMS, VDR			TYMS	RARA, RXRA, SHMT1, SIRT5, TOP1, TYMS						
Anti-Obesity	3<br>CA2, PNLIP, FASN	3<br>CA2, PNLIP, FASN	3<br>CA2, PNLIP, FASN	3<br>CA2, PNLIP, FASN	3<br>CA2, PNLIP, FASN	1<br>CA2	2<br>CA2, FASN	3<br>CA2, PNLIP, FASN	1<br>FASN	1<br>PNLIP	1<br>FASN	1<br>CA2	3<br>CA2, PNLIP, FASN	3
Antirheumatism	6<br>DHFR, C1R, HPRT1, FCGR3B, ACAT1, PPARG	7<br>DHFR, TNF, C1R, HPRT1, FCGR3B, ACAT1, PPARG	7<br>DHFR, TNF, C1R, HPRT1, FCGR3B, ACAT1, PPARG	6<br>DHFR, DHODH, C1R, HPRT1, FCGR3B, ACAT1	7<br>DHFR, TNF, C1R, HPRT1, FCGR3B, ACAT1, PPARG	1<br>DHFR	5<br>DHFR, TNFRSF1B, TNF, HPRT1, ACAT1	7<br>DHFR, TNF, C1R, HPRT1, FCGR3B, ACAT1, PPARG	3<br>DHFR, TNFRSF1B, PPARG	2<br>DHFR, ACAT1	1<br>PPARG	3<br>TNFRSF1B, FCGR3B, PPARG	7<br>DHFR, DHODH, C1R, HPRT1, FCGR3B, ACAT1, PPARG	9
Antithrombus	7<br>PDE10A, PPARD, F2, SELP, F10, NOS2, SERPINC1	7<br>PDE10A, PPARD, F2, SELP, F10, NOS2, SERPINC1	7<br>PDE10A, PPARD, F2, SELP, F10, NOS2, SERPINC1	7<br>PDE10A, PPARD, F2, SELP, F10, NOS2, SERPINC1	8<br>PDE10A, PPARD, F2, SERPIND1, SELP, F10, NOS2, SERPINC1	6<br>PDE10A, PPARD, F2, SELP, NOS2, SERPINC1	8<br>PDE10A, PPARD, F2, SERPIND1, SELP, F10, NOS2, SERPINC1	7<br>PDE10A, PPARD, F2, SELP, F10, NOS2, SERPINC1	5<br>PDE10A, F2, SERPIND1, NOS2, SERPINC1	4<br>F2, F10, NOS2, SERPINC1	5<br>PDE10A, F2, SERPIND1, NOS2, SERPINC1	2<br>F2, NOS2	7<br>PDE10A, PPARD, F2, SELP, F10, NOS2, SERPINC1	8
Bronchodilator	9<br>NR3C1, PAH, MAPK1, PDE4B, PDE5A, NR3C2, PGR, PDE7A, PDE4D	9<br>NR3C1, PAH, TNF, PDE4B, PDE5A, NR3C2, PGR, PDE7A, PDE4D	9<br>NR3C1, PAH, NR3C2, TNF, PDE4B, PDE5A, PGR, PDE7A, PDE4D	8<br>NR3C1, PAH, NR3C2, PDE4B, PDE5A, PGR, PDE7A, PDE4D	10<br>NR3C1, PAH, MAPK1, TNF, PDE4B, PDE5A, NR3C2, PGR, PDE7A, PDE4D	7<br>NR3C1, PAH, PDE4B, PDE5A, PGR, PDE7A, PDE4D	9<br>NR3C1, PAH, MAPK1, TNF, PDE4B, PDE5A, NR3C2, PDE7A, PDE4D	8<br>NR3C1, PAH, PDE5A, NR3C2, TNF, PGR, PDE7A, PDE4D	2<br>PAH, PDE4D	6<br>NR3C1, PAH, PDE5A, PDE4B, PGR, PDE4D	2<br>PDE5A, PDE4D	1<br>PDE4D	7<br>NR3C1, PAH, PDE5A, NR3C2, PGR, PDE7A, PDE4D	10
Fibrinolysis	5<br>ITGB3, F2, F10, PPARG, SERPINC1	5<br>ITGB3, F2, F10, PPARG, SERPINC1	6<br>ITGB3, F2, ITGA2B, F10, PPARG, SERPINC1	4<br>ITGB3, F2, F10, SERPINC1	6<br>ITGB3, F2, ITGA2B, F10, PPARG, SERPINC1	3<br>ITGB3, F2, SERPINC1	4<br>ITGB3, F2, F10, SERPINC1	6<br>ITGB3, F2, ITGA2B, F10, PPARG, SERPINC1	4<br>ITGB3, F2, PPARG, SERPINC1	4<br>ITGB3, F2, F10, SERPINC1	3<br>F2, PPARG, SERPINC1	2<br>F2, PPARG	6<br>ITGB3, F2, ITGA2B, F10, PPARG, SERPINC1	6
Hypoglycemia	8<br>IGF1R, INSR, CFTR, CTSD, DPP4, IDE, AMY2A, PPARG	8<br>IGF1R, INSR, CFTR, CTSD, DPP4, IDE, AMY2A, PPARG	8<br>IGF1R, INSR, CFTR, CTSD, DPP4, IDE, AMY2A, PPARG	7<br>IGF1R, INSR, CFTR, CTSD, DPP4, IDE, AMY2A	8<br>IGF1R, INSR, CFTR, CTSD, DPP4, IDE, AMY2A, PPARG	7<br>IGF1R, INSR, CFTR, CTSD, DPP4, IDE, AMY2A	6<br>INSR, CFTR, CTSD, DPP4, IDE, AMY2A	8<br>IGF1R, INSR, CFTR, CTSD, DPP4, IDE, AMY2A, PPARG	7<br>IGF1R, CFTR, CTSD, DPP4, IDE, INSR, PPARG	5<br>INSR, CFTR, CTSD, DPP4, IDE	7<br>IGF1R, CFTR, CTSD, DPP4, IDE, INSR, PPARG	6<br>INSR, CFTR, CTSD, DPP4, IDE, PPARG	8<br>IGF1R, INSR, CFTR, CTSD, DPP4, IDE, AMY2A, PPARG	8
Immunomodulatory agent	15<br>CD247, ITGAV, ITGB3, C3, CD1A, CD4, FCER1A, PPIA, ELANE, C1R, ICAM1, ITGAL, FCGR3B, IL2RA, IL2RB	16<br>CD247, ITGAV, ITGB3, C3, CD1A, CD4, FCER1A, PPIA, ELANE, TNF, C1R, ICAM1, ITGAL, FCGR3B, IL2RA, IL2RB	15<br>CD247, ITGAV, ITGB3, C3, CD1A, CD4, FCER1A, PPIA, TNF, C1R, ICAM1, ITGAL, FCGR3B, IL2RA, IL2RB	15<br>CD247, ITGAV, ITGB3, C3, CD1A, CD4, FCER1A, PPIA, ELANE, C1R, ICAM1, ITGAL, FCGR3B, IL2RA, IL2RB	15<br>CD247, ITGAV, ITGB3, C3, CD1A, CD4, FCER1A, PPIA, TNF, C1R, ICAM1, ITGAL, FCGR3B, IL2RA, IL2RB	12<br>CD247, ITGAV, ITGB3, C3, CD1A, CD4, FCER1A, PPIA, ICAM1, ITGAL, IL2RA, IL2RB	10<br>CD247, ITGAV, ITGB3, C3, CD1A, CD4, FCER1A, PPIA, TNFRSF1B, TNF	16<br>CD247, ITGAV, ITGB3, C3, CD1A, CD4, FCER1A, PPIA, ELANE, TNF, C1R, ICAM1, ITGAL, FCGR3B, IL2RA, IL2RB	8<br>ITGAV, ITGB3, C3, CD1A, CD4, FCER1A, PPIA, TNFRSF1B	8<br>CD247, ITGAV, ITGB3, C3, CD1A, CD4, FCER1A, PPIA	5<br>C3, CD1A, CD4, FCER1A, PPIA	9<br>C3, CD1A, CD4, FCER1A, PPIA, TNFRSF1B, FCGR3B, IL2RA, IL2RB	15<br>CD247, ITGAV, ITGB3, C3, CD1A, CD4, FCER1A, PPIA, C1R, ICAM1, ITGAL, FCGR3B, IL2RA, IL2RB, C4A	18

[a] The ingredient (drug)-target interaction pairs for each Yadanzi's pharmacological activity, e.g. anticancer, were determined by superimposing non-redundant putative targets of Yadanzi ingredients predicted by reverse docking approach against activity-associated proteins collected from on-line resources like KEGG and literatures.

Table S2. The binding affinity comparison between Yadanzi ingredients and their corresponding marketing drugs when they bind to the same protein target.

Targets	PDB_ID	Drugs			Ingredients		
		Name	MM/GBVI (kcal/mol)	Affinity(pki)	Name	MM/GBVI (kcal/mol)	Affinity(pki)
ABL1	3EG3	Imatinib	-19.554	7.326	bruceine B	-17.693	5.437
AKR1B1	1US0	Sulindac	-24.997	6.462	bruceoside A	-29.122	8.013
					bruceantinol	-21.496	5.815
AR	2AX6	Flutamide	-21.536	6.933	javanicin	-25.747	9.32
ATIC	1P4R	Pemetrexed	-22.102	6.225	bruceine C	-41.855	13.78
					bruceoside B	-34.52	12.026
					bruceoside A	-27.845	8.441
					bruceine B	-25.856	7.285
					bruceantinol	-23.583	7.686
					javanicin	-19.428	6.76
					bruceine I	-18.234	8.441
BRAF	1UWH	Sorafenib	-27.164	7.196	bruceoside B	-33.072	8.225
					bruceine C	-27.343	6.303
					Bruceantinol	-25.173	8.021
					javanicin	-22.758	6.711
					bruceoside A	-22.544	7.215
					bruceine I	-20.339	5.733
CHD1	2B2W	Epirubicin	-26.73	12.638	bruceantinol	-31.501	17.845
					javanicin	-28.987	8.337
					brusatol	-28.967	7.825
CSF1R	2I1M	Imatinib	-26.86	9.335	bruceine C	-24.22	6.837
DHFR	1KMV	Methotrexate	-35.952	9.568	bruceantinol	-27.828	11.028
					javanicin	-23.092	9.023
DHODH	3ZWT	Leflunomide	-19.635	7.462	javanicin	-25.343	9.835
EGFR	1M17	Erlotinib	-15.565	8.051	bruceine C	-26.689	8.727
		Lapatinib	-25.746	9.897	bruceine E	-26.019	8.493
		Gefitinib	-25.655	8.474	bruceoside A	-25.223	10.02
					bruceoside B	-24.587	6.695
					bruceoside E	-23.026	10.697
					bruceantin	-22.556	6.988
					bruceine D	-21.808	7.058
					bruceantinol	-19.816	6.735
					bruceine G	-18.179	6.11
					javanicin	-18.037	6.494
ERBB2	3PP0	Lapatinib	-38.773	12.327	bruceantin	-25.245	7.159
					bruceoside B	-23.663	7.2
					bruceine C	-22.998	6.846
ESRRG	2E2R	Diethylstilbestrol	-14.958	6.175	bruceine C	-32.183	9.595
F2	2BVR	Suramin	-47.664	10.746	bruceine C	-49.037	12.206
					bruceoside B	-42.289	11.459
					bruceantinol	-40.149	10.093
					bruceine B	-38.365	11.515
					bruceoside E	-33.542	11.704
					javanicin	-31.446	9.505
FDPS	2QIS	Pamidronate	-26.141	8.684	bruceine I	-22.067	7.191
HMGCR	3CCZ	Lovastatin	-22.954	7.297	bruceantinol	-26.048	8.279
					bruceine C	-24.338	7.102
					bruceine B	-22.589	7.596

IMPDH1	1JCN	Mycophenolate mofetil	-27.377	9.48	bruceantinol	-31.491	10.768
IMPDH2	1NF7	Mycophenolate mofetil	-26.874	8.168	bruceoside A	-33.149	11.043
					bruceine C	-29.788	11.225
					bruceine G	-25.999	8.35
					bruceantinol	-25.155	11.124
					bruceantin	-23.597	9.853
					bruceine I	-22.991	9.258
					bruceine B	-21.696	10.705
					javanicin	-20.404	7.586
ITGAL	2ICA	Lovastatin	-15.959	6.715	javanicin	-20.906	8.215
					bruceine B	-16.38	7.09
					bruceine E	-12.035	10.705
KDR	2XIR	Sorafenib	-35.357	11.256	bruceine C	-40.469	8.987
					bruceoside B	-38.596	9.878
					bruceoside E	-29.677	10.098
					bruceine I	-19.345	7.162
					bruceantinol	-19.224	8.199
KIT	3G0E	Sorafenib	-25.827	8.422	bruceoside E	-19.121	10.317
					bruceine B	-18.834	7.488
MMP1	1HFC	Marimastat	-17.288	5.931	bruceoside E	-291.2	21.28
					bruceine C	-137.49	7.732
					bruceine E	-38.881	11.529
					bruceantin	-23.674	6.991
					javanicin	-21.043	5.732
					bruceoside B	-20.976	9.208
MMP10	1Q3A	Marimastat	-25.978	6.607	bruceoside E	-342.849	20.609
					bruceine C	-149.387	10.908
					bruceine E	-52.825	12.198
					javanicin	-35.746	5.315
MMP12	1Y93	Marimastat	-31.953	5.251	bruceine C	-125.984	6.629
					bruceoside A	-34.135	5.216
					bruceoside B	-32.39	5.842
MMP13	3ZXH	Marimastat	-20.122	4.53	javanicin	-30.856	4.913
					bruceine C	-25.679	6.011
					brusatol	-22.997	8.924
MMP2	1HOV	Marimastat	-25.879	4.451	bruceoside E	-542.643	33.914
					bruceine E	-71.895	17.592
					bruceantin	-23.238	4.962
					bruceantinol	-18.406	4.89
MMP3	1HY7	Marimastat	-33.438	7.214	bruceine E	-45.35	11.473
					bruceine C	-35.907	5.487
MMP7	2Y6D	Marimastat	-32.87	6.036	bruceine E	-50.418	11.103
					javanicin	-29.123	8.386
					brusatol	-23.929	6.041
MMP8	1I76	Marimastat	-19.645	6.376	bruceine E	-50.46	12.627
					bruceine D	-29.848	6.914
					bruceantin	-24.563	6.066
					brusatol	-24.504	3.618
					bruceine I	-23.805	6.28
					bruceine G	-16.199	8.078
MMP9	2OVX	Marimastat	-18.499	6.548	javanicin	-19.537	6.125

NOS2	3E7G	Dexamethasone	-17.896	6.151	bruceine C	-142.23	8.392
					bruceoside E	-52.52	11.842
					bruceoside B	-31.241	7.939
					bruceoside A	-28.302	8.469
					bruceantinol	-24.676	6.984
					bruceine I	-21.79	7.755
					bruceine B	-18.856	6.314
NR3C1	3K22	Flunisolide	-22.44	9.224	javanicin	-20.993	7.117
PDPK1	1H1W	7-Hydroxystaurosporine*	-33.622	9.67	bruceantin	-29.895	9.698
					bruceoside B	-29.752	9.59
					bruceine E	-26.645	7.847
					bruceine I	-24.293	7.759
					bruceantinol	-22.751	9.649
					bruceine G	-22.683	9
					bruceine C	-22.248	7.008
					brusatol	-21.152	8.054
					bruceine D	-20.71	7.235
					bruceine B	-19.655	9.113
PGR	1SQN	Megestrol	-26.269	8.331	javanicin	-25.565	7.017
PLA2G2A	1KVO	Suramin	-47.158	10.526	bruceoside E	-172.629	27.043
					bruceine C	-95.33	8.452
					bruceine E	-27.312	9.287
PNP	3BGS	Cladribine	-25.842	7.13	bruceoside A	-35.97	10.663
					bruceantinol	-24.923	7.816
					bruceoside E	-19.317	10.108
POLB	3OGU	Cytarabine	-17.189	5.245	bruceoside E	-39.394	13.394
					bruceoside A	-32.743	7.781
					bruceine C	-22.135	5.771
					bruceantinol	-20.806	6.981
PPARD	3TKM	Sulindac	-17.377	4.658	javanicin	-18.274	6.234
RARA	3A9E	Alitretinoin	-35.486	9.393	javanicin	-22.049	7.892
SIRT5	3RIY	Suramin	-45.281	7.981	javanicin	-22.009	9.668
TOP1	1K4T	Irinotecan	-27.813	7.185	bruceoside A	-34.469	10.568
					bruceantinol	-31.527	7.669
					bruceoside B	-30.118	8.146
					bruceine I	-30.026	7.121
					brusatol	-28.835	7.809
					bruceine C	-26.381	7.62
					javanicin	-25.298	7.982
					bruceine D	-23.311	8.675
					bruceine E	-22.543	13.232
					bruceine B	-22.153	6.336
TYMS	1HVY	Raltitrexed	-31.948	9.098	bruceine C	-30.566	9.031
					bruceantinol	-26.186	8.215
					javanicin	-24.629	7.894
VDR	3B0T	Calcitriol	-34.923	10.175	javanicin	-24.512	11.345

* A research drug of PDPK1.

For each protein target, same active pocket site was adopted for binding affinity comparison between an ingredient-drug pair. The docking was demonstrated using the Ligand Explorer model (LigX) module of the commercial software MOE with the parameters of: Receptor: Receptor+Solvent, Rescoring 1: London dG, Retain: 30, Refinement: Forcefield (Amber99). Solvation effects were calculated using the reaction field functional form for the electrostatic energy term and a dielectric constant of 4. The lower MM/GBVI suggests the higher binding affinity, while the higher pki indicates better binding affinity.

Table S3. The list of anticancer protein targets, their corresponding marketing drugs and the Yadanzi ingredients that were predicted to bind to these targets.

Targets	Drugs	Ingredients *
ABL1	Dasatinib, Nilotinib, Imatinib	A; B; C; D; E; F; G; H; I; J; K; L; M
AKR1B1	Nilutamide, Sulindac	A; B; C; D; F; G; H; I; J; K; L; M
AR	Oxandrolone, Nilutamide, Bicalutamide, Flutamide	C; D; H; I; J
ATIC	Pemetrexed	B; C; D; E; F; G; H; I; J; K; L; M
BRAF	Sorafenib	A; B; C; E; F; G; H; I; J; K; L; M
C1R	Cetuximab#	B; C; D; H; I; J; K
CHD1	Epirubicin	A; B; C; D; E; F; G; H; I; J; K; L; M
CSF1R	Sunitinib	B; C; D; F; G; H; I; J; K
DCK	Fludarabine	B; C; D; G; H; I; J; K
DHFR	Pemetrexed, Trimetrexate	A; B; C; D; F; G; H; I; J; K; L
DHODH	Leflunomide	H; I
EGFR	Erlotinib, Lapatinib, Gefitinib	A; B; C; D; E; F; G; H; I; J; K; L; M
ERBB2	Trastuzumab#	B; C; D; E; F; G; H; I; J; K
ESR1	Chlorotrianisene, Toremifene, Estrone, Tamoxifen, Fulvestrant, Fluoxymesterone, Estramustine, Diethylstilbestrol	A; B; C; D; F; G; H; I; K
ESRRG	Diethylstilbestrol	A; B; C; D; E; F; G; H; I; J; K; L
F2	Suramin	A; B; C; D; E; F; G; H; I; J; K; L; M
FDPS	Pamidronate	B; C; D; E; F; G; H; I; J; K; L
FSHR	Suramin	A; C; D; F; H; I; J; K; M
GART	Pemetrexed	A; B; C; D; E; F; G; H; I; J; K; L; M
GSR	Carmustine	B; C; D; F; G; H; I; J; K
HDAC8	Vorinostat	B; C; D; I; J
HMGCR	Lovastatin	A; B; C; D; E; F; G; H; I; J; K; L; M
HPRT1	Azathioprine	B; C; D; F; H; I; J; K
IMPDH1	Mycophenolic acid, Mycophenolate	L
IMPDH2	Mycophenolic acid, Mycophenolate	A; B; C; D; F; G; H; I; J; K; L; M
ITGAL	Lovastatin	B; C; D; G; H; I; J; K
KDR	Sunitinib, Sorafenib	A; B; C; D; E; F; G; H; L
KIT	Sorafenib, Imatinib, Sunitinib	A; B; C; D; E; H; I; K
LDLR	Porfimer	B; C; D; F; H; I; J; L
MAPK1	Arsenic trioxide#	B; F; K
MMP1	Marimastat	A; B; C; D; E; F; G; H; I; J; K; L
MMP10	Marimastat	A; B; C; D; F; G; H; I; J; K
MMP12	Marimastat	A; B; C; D; E; F; G; H; I; J; K; L; M
MMP13	Marimastat	F; I; K
MMP2	Marimastat	A; B; C; D; G; H; I; J; K; L
MMP3	Marimastat	B; C; D; F; G; H; I; J; K; L
MMP7	Marimastat	B; C; H; I; K
MMP8	Marimastat	B; C; D; G; H; I; J; K

MMP9	Marimastat	I; J
NOS2	Dexamethasone	A; B; C; D; E; F; G; H; I; J; K; L; M
NR3C1	Megestrol, Prednisone, Prednisolone, Fluoxymesterone, Dexamethasone, Flunisolide	C; D; H; I; J; K
PGR	Megestrol	B; C; D; G; H; I; J; K; L
PLA2G2A	Suramin,	A; B; C; D; F; G; H; I; J; K; L
PNP	Cladribine	A; B; C; D; F; G; H; I; J; K; L; M
POLB	Cytarabine	A; B; C; D; E; F; G; H; I; J; K; L; M
PPARD	Sulindac	A; B; C; D; F; G; H; I; J; K;
RARA	Isotretinoin, Alitretinoin	B; C; D; E; F; H; K; L;
RXRA	Alitretinoin	A; B; C; D; F; G; H; I; J; K; L; M
SHMT1	Mimosine	B; C; D; F; H; I; J; K
SIRT5	Suramin	B; C; D; F; G; H; I; J; K
TOP1	Topotecan, Irinotecan	A; B; C; D; E; F; G; H; I; J; K; L; M
TUBG1	Vinblastine	E; K
TYMS	Raltitrexed, Floxuridine, Gemcitabine, Fluorouracil, Pemetrexed, Leucovorin, Capecitabine	B; C; D; F; G; H; I; J; K; L
VDR	Calcitriol	H; I; J
PDPK1	7-Hydroxystaurosporine	B; C; D; E; F; G; H; I; J; K; L

* bruceoside_e: A, bruceine_b: B, bruceine_d: C, bruceine_e: D, bruceoside_b: E, bruceine_c: F, bruceantin: G, bruceine_i: H, javanicin: I, bruceine_g: J, brusatol: K, bruceantinol: L, bruceoside_a: M.

# The marked drugs are either antibodies or mineral medicines, which were not applicable for docking analysis and pharmacophore analysis.

Table S4. The consensus pharmacophore analysis of marketing drugs and Yadanzi ingredients when they bind to the common protein targets.

Targets	Drugs Number	Ingredients Number	Pharmacophore Feature Groups		
			Group 1	Group 2	Group 3
ABL1	3	13	92.31%	92.31%	92.31%
AKR1B1	2	12	66.67%	66.67%	0.00%
AR	4	5	80.00%	60.00%	60.00%
ATIC	1	12	66.67%	58.33%	0.00%
BRAF	1	12	66.67%	41.67%	0.00%
CHD1	1	13	61.54%	61.54%	61.54%
CSF1R	1	9	44.44%	44.44%	55.56%
DCK	1	8	100.00%	25.00%	37.50%
DHFR	2	11	90.91%	0.00%	0.00%
DHODH	1	2	100.00%	100.00%	50.00%
EGFR	3	13	84.62%	76.92%	69.23%
ESR1	8	9	100.00%	77.78%	0.00%
ESRRG	1	12	58.33%	50.00%	41.67%
F2	1	13	61.54%	69.23%	53.85%
FDPS	1	11	90.91%	54.55%	0.00%
FSHR	1	9	88.89%	88.89%	44.44%
GART	1	13	76.92%	0.00%	0.00%
GSR	1	9	77.78%	44.44%	0.00%
HDAC8	1	5	100.00%	60.00%	80.00%
HMGCR	1	13	76.92%	69.23%	61.54%
HPRT1	1	8	87.50%	50.00%	50.00%
IMPDH1	2	1	100.00%	100.00%	100.00%
IMPDH2	2	12	75.00%	50.00%	0.00%
ITGAL	1	8	75.00%	50.00%	0.00%
KDR	2	9	100.00%	66.67%	55.56%
KIT	3	8	75.00%	50.00%	37.50%
LDLR	1	8	100.00%	62.50%	62.50%
MMP1	1	12	66.67%	66.67%	66.67%
MMP10	1	10	80.00%	70.00%	50.00%
MMP12	1	13	69.23%	61.54%	53.85%
MMP13	1	3	66.67%	66.67%	33.33%
MMP2	1	10	60.00%	60.00%	40.00%
MMP3	1	10	80.00%	80.00%	70.00%
MMP7	1	5	80.00%	40.00%	40.00%
MMP8	1	8	12.50%	0.00%	0.00%
MMP9	1	2	100.00%	100.00%	100.00%

NOS2	1	13	69.23%	69.23%	61.54%
NR3C1	6	6	100.00%	100.00%	0.00%
PGR	1	9	88.89%	77.78%	55.56%
PLA2G2A	1	11	81.82%	81.82%	54.55%
PNP	1	12	83.33%	66.67%	75.00%
POLB	1	13	100.00%	76.92%	69.23%
PPARD	1	10	70.00%	60.00%	0.00%
RARA	2	8	87.50%	62.50%	0.00%
RXRA	1	8	83.3%	0.00%	0.00%
SHMT1	1	8	87.50%	75.00%	62.50%
SIRT5	1	9	88.89%	77.78%	55.56%
TOP1	2	13	69.23%	69.23%	61.54%
TUBG1	1	2	100.00%	100.00%	100.00%
TYMS	7	10	60.00%	30.00%	60.00%
VDR	1	3	100.00%	66.67%	66.67%
PDPK1	1	11	90.91%	81.82%	72.73%

The target-drug-ingredients relations were acquired from the Table S5. The pharmacophore feature groups were determined using the Pharmacophore module of the Molecular Operating Environment (MOE) software. Only those pharmacophore groups that contains at least one marketing drugs were chosen for consensus pharmacophore analysis. For each pharmacophore group, the consensus of pharmacophore was measured by calculating the number of ingredients that contain this pharmacophore group against all ingredients that bind to the target protein (refer to the supplementary method section). The consensus of the top 3 consistent pharmacophore groups was given here.

Table S5. The list of Yadanzi ingredient-target pairs that were estimated to have better binding affinity than their corresponding protein-drug interactions.

Targets	PDB_ID	Drugs			Ingredients		
		Name	MM/GBVI (kcal/mol)	Affinity (pki)	Name	MM/GBVI (kcal/mol)	Affinity (pki)
AKR1B1	1US0	Sulindac	-24.997	6.462	Bruceoside A	-29.122	8.013
ATIC	1P4R	Pemetrexed	-22.102	6.225	Bruceine C	-41.855	13.78
					Bruceoside B	-34.52	12.026
					Bruceoside A	-27.845	8.441
BRAF	1UWH	Sorafenib	-27.164	7.196	Bruceoside B	-33.072	8.225
CHD1	2B2W	Epirubicin	-26.73	12.638	Bruceantinol	-31.501	17.845
					Javanicin	-28.987	8.337
					Brusatol	-28.967	7.825
ESRRG	2E2R	Diethylstilbestro l	-14.958	6.175	Bruceine C	-32.183	9.595
F2	2BVR	Suramin	-47.664	10.746	Bruceine C	-49.037	12.206
IMPDH1	1JCN	Mycophenolate mofetil	-27.377	9.48	Bruceantinol	-31.491	10.768
					Bruceoside A	-33.149	11.043
					Bruceine C	-29.788	11.225
MMP1	1HFC	Marimastat	-17.288	5.931	Bruceoside E	-291.2	21.28
					Bruceine C	-137.49	7.732
					Bruceine E	-38.881	11.529
MMP10	1Q3A	Marimastat	-25.978	6.607	Bruceoside E	-342.849	20.609
					Bruceine C	-149.387	10.908
					Bruceine E	-52.825	12.198
MMP12	1Y93	Marimastat	-31.953	5.251	Bruceine C	-125.984	6.629
MMP2	1HOV	Marimastat	-25.879	4.451	Bruceoside E	-542.643	33.914
					Bruceine E	-71.895	17.592
MMP3	1HY7	Marimastat	-33.438	7.214	Bruceine E	-45.35	11.473
MMP7	2Y6D	Marimastat	-32.87	6.036	Bruceine E	-50.418	11.103
					Javanicin	-29.123	8.386
MMP8	1I76	Marimastat	-19.645	6.376	Bruceine E	-50.46	12.627
					Bruceine D	-29.848	6.914
NOS2	3E7G	Dexamethason e	-17.896	6.151	Bruceine C	-142.23	8.392
					Bruceoside E	-52.52	11.842
					Bruceoside B	-31.241	7.939
					Bruceoside A	-28.302	8.469
PLA2G2A	1KVO	Suramin	-47.158	10.526	Bruceoside E	-172.629	27.043
PNP	3BGS	Cladribine	-25.842	7.13	Bruceoside A	-35.97	10.663
POLB	3OGU	Cytarabine	-17.189	5.245	Bruceoside E	-39.394	13.394
					Bruceoside A	-32.743	7.781
PPARD	3TKM	Sulindac	-17.377	4.658	Javanicin	-18.274	6.234
TOP1	1K4T	Irinotecan	-27.813	7.185	Bruceoside A	-34.469	10.568
					Bruceantinol	-31.527	7.669
					Bruceoside B	-30.118	8.146
					Brusatol	-28.835	7.809

For each therapeutic target, same active pocket site was adopted for binding affinity comparison between an ingredient and drug pair. The docking was demonstrated and refined using the commercial software MOE with the parameters of Receptor: Receptor+Solvent, Rescoring 1: London dG, Retain: 30, Refinement: Forcefield (MMFF94x). The complete result is given in the Table S4.

Table S6. The list of the enriched cancer pathways that the Yadanzi ingredients are likely associated with.

Cancer Pathway	Count	Percentage (%)*	Fold Enrichment	P value	Putative targets
hsa05213:Endometrial cancer	18	2.99	2.92	5.67E-05	EGFR, PIK3CG, HRAS, BRAF, MAP2K1, MAP2K2, GRB2, ERBB2, CTNNA1, PTEN, CTNNB1, MAPK1, PDPK1, CASP9, GSK3B, SOS1, APC, AKT2
hsa05219:Bladder cancer	14	2.32	2.81	7.66E-04	EGFR, HRAS, BRAF, MAP2K1, MAP2K2, ERBB2, MMP9, DAPK2, MMP2, MMP1, DAPK1, MAPK1, TYMP, MDM2
hsa05223:Non-small cell lung cancer	17	2.82	2.65	3.39E-04	EGFR, PIK3CG, FHIT, HRAS, MAP2K1, BRAF, MAP2K2, GRB2, RXRA, ERBB2, CDK6, PRKCB, MAPK1, PDPK1, CASP9, SOS1, AKT2
hsa05220:Chronic myeloid leukemia	23	3.81	2.59	3.25E-05	PIK3CG, HRAS, CTBP1, CTBP2, BCR, MAP2K1, BRAF, MAP2K2, GRB2, TGFBR1, CBL, SMAD4, CDK6, BCL2L1, PTPN11, MAPK1, CBLB, CDKN1B, SOS1, MDM2, ABL1, CRK, AKT2
hsa05215:Prostate cancer	26	4.31	2.46	2.18E-05	FGFR1, HRAS, GRB2, ERBB2, PTEN, CTNNB1, IGF1R, PDPK1, CASP9, INS, SOS1, AKT2, EGFR, PIK3CG, AR, HSP90AA1, BRAF, MAP2K1, MAP2K2, CDK2, MAPK1, CDKN1B, GSK3B, MDM2, MTOR, GSTP1
hsa05210:Colorectal cancer	24	3.98	2.41	7.19E-05	EGFR, PIK3CG, MSH6, MAP2K1, BRAF, MSH2, GRB2, TGFBR1, SMAD4, SMAD2, MAPK10, CTNNB1, MAPK1, IGF1R, CASP3, RAC2, CASP9, RAC3, GSK3B, SOS1, RAC1, MAPK8, APC, AKT2
hsa05216:Thyroid cancer	8	1.33	2.33	4.80E-02	MAPK1, HRAS, MAP2K1, BRAF, MAP2K2, RXRA, PPARG, CTNNB1
hsa05214:Glioma	17	2.82	2.28	2.13E-03	EGFR, PIK3CG, HRAS, BRAF, MAP2K1, MAP2K2, GRB2, CDK6, PTEN, PRKCB, IGF1R, MAPK1, SOS1, MDM2, MTOR, CALM1, AKT2
hsa05212:Pancreatic cancer	19	3.15	2.23	1.40E-03	EGFR, PIK3CG, MAP2K1, BRAF, ERBB2, TGFBR1, SMAD4, CDK6, SMAD2, BCL2L1, MAPK10, CDC42, MAPK1, RAC2, CASP9, RAC3, RAC1, MAPK8, AKT2
hsa05221:Acute myeloid leukemia	15	2.49	2.18	6.45E-03	PIK3CG, HRAS, PPARD, BRAF, MAP2K1, MAP2K2, GRB2, PIM1, KIT, MAPK1, EIF4EBP1, SOS1, RARA, MTOR, AKT2
hsa05211:Renal cell carcinoma	15	2.49	1.81	3.25E-02	PIK3CG, HRAS, BRAF, MAP2K1, MAP2K2, GRB2, EGLN1, PTPN11, MAPK1, CDC42, SOS1, PAK4, RAC1, CRK, AKT2
hsa05200:Pathways in cancer	70	11.6	1.8	6.10E-07	PPARD, HRAS, MMP9, PPARG, MMP2, PTEN, MMP1, CTNNB1, CDC42, CASP3, CASP9, RHOA, RARA, NOS2, FGF1, AKT2, EGFR, PIK3CG, CTBP1, AR, HSP90AA1, CTBP2, BCR, BRAF, RXRA, SKP2, CDK6, DAPK2, CTNNA1, CDK2, DAPK1, PRKCB, MAPK1, MDM2, MAPK8, GSTP1, ITGA2B, CKS1B, TRAF2, FGFR1, GRB2, ERBB2, KITLG, EGLN1, BCL2L1, KIT, IGF1R, PTK2, RAC2, RAC3, SOS1, ITGAV, RAC1, APC, CSF1R, MSH6, MAP2K1, MSH2, MAP2K2, TGFBR1, CBL, SMAD4, SMAD2, MAPK10, CBLB, CDKN1B, GSK3B, MTOR, ABL1, CRK
hsa05222:Small cell lung cancer	17	2.82	1.71	3.53E-02	PIK3CG, FHIT, CKS1B, TRAF2, RXRA, SKP2, CDK6, BCL2L1, PTEN, CDK2, PTK2, CDKN1B, CASP9, ITGAV, NOS2, AKT2, ITGA2B
hsa05218:Melanoma	14	2.32	1.66	7.08E-02	PIK3CG, EGFR, FGFR1, HRAS, BRAF, MAP2K1, MAP2K2, CDK6, PTEN, IGF1R, MAPK1, MDM2, FGF1, AKT2

* Percentage out of the 603 targets annotated in the KEGG pathways.
